# Iris metastasis preceding diagnosis of gastric signet ring cell adenocarcinoma: a case report

**DOI:** 10.1186/s12886-018-0795-1

**Published:** 2018-05-25

**Authors:** Tadanobu Yoshikawa, Kimie Miyata, Tokiko Nakai, Chiho Ohbayashi, Miki Kaneko, Nahoko Ogata

**Affiliations:** 1 Department of Ophthalmology, Nara Medical University School of Medicine, 840 Shijo-cho, Kashihara, Nara, 634-8522 Japan; 2 Department of Diagnostic Pathology, Nara Medical University School of Medicine, Nara, Japan; 3 Department of Internal Medicine, Kokuho Central Hospital, Nara, Japan

**Keywords:** Iritis, Signet ring cell, Glaucoma, Iris metastasis, Choroidal metastasis

## Abstract

**Background:**

A case of iris metastasis preceding the diagnosis of gastric signet ring cell adenocarcinoma is very rare. To report the findings in a patient who presented with an iris tumor that was later identified to have metastasized from a gastric signet ring cell adenocarcinoma.

**Case presentation:**

A-74-year-old woman presented with visual disturbance and an increased intraocular pressure (IOP) in the right eye. She had no history of systemic cancer. She was initially diagnosed with acute iritis from diabetes mellitus and secondary glaucoma. She underwent trabeculectomy because of the uncontrolled IOP. After the IOP was controlled, she presented thick iris with corectopia, iris hemorrhage, and white, frog spawn-like mass resembling fibrin in the anterior chamber. An analysis of an iris biopsy suggested that the iris mass was an adenocarcinoma. Examination by esophagogastroduodenoscopy revealed advanced gastric signet ring cell adenocarcinoma as the primary source for the iris tumor.

**Conclusions:**

We recommend that patients with acute iritis with atypical iris mass resembling fibrin and secondary glaucoma should be examined comprehensively for systemic tumors.

## Background

The uvea is the most common site of ophthalmic metastatic lesions from systemic tumors because of its high blood flow. The common uveal tumors are metastasis from breast and lung tumors [1], and iris metastasis from the gastrointestinal tract are rare [1]. The anatomic location of the uveal metastasis is most commonly found in the choroid [1], and there have been only 4 cases of iris metastasis from gastric cancer [1–3] and only one case of a gastric signet ring cell adenocarcinoma [2].

A diagnosis of iris metastasis in patients without a detectable mass and a history of systemic cancer is very difficult because the signs and symptoms are very similar to that of inflammatory uveitis. Furthermore the manifestations of iris metastasis from gastric adenocarcinoma have not been fully determined.

We report a case of an iris metastasis that was identified before the diagnosis of gastric signet ring cell adenocarcinoma.

## Case presentation

**Fig. 1:**
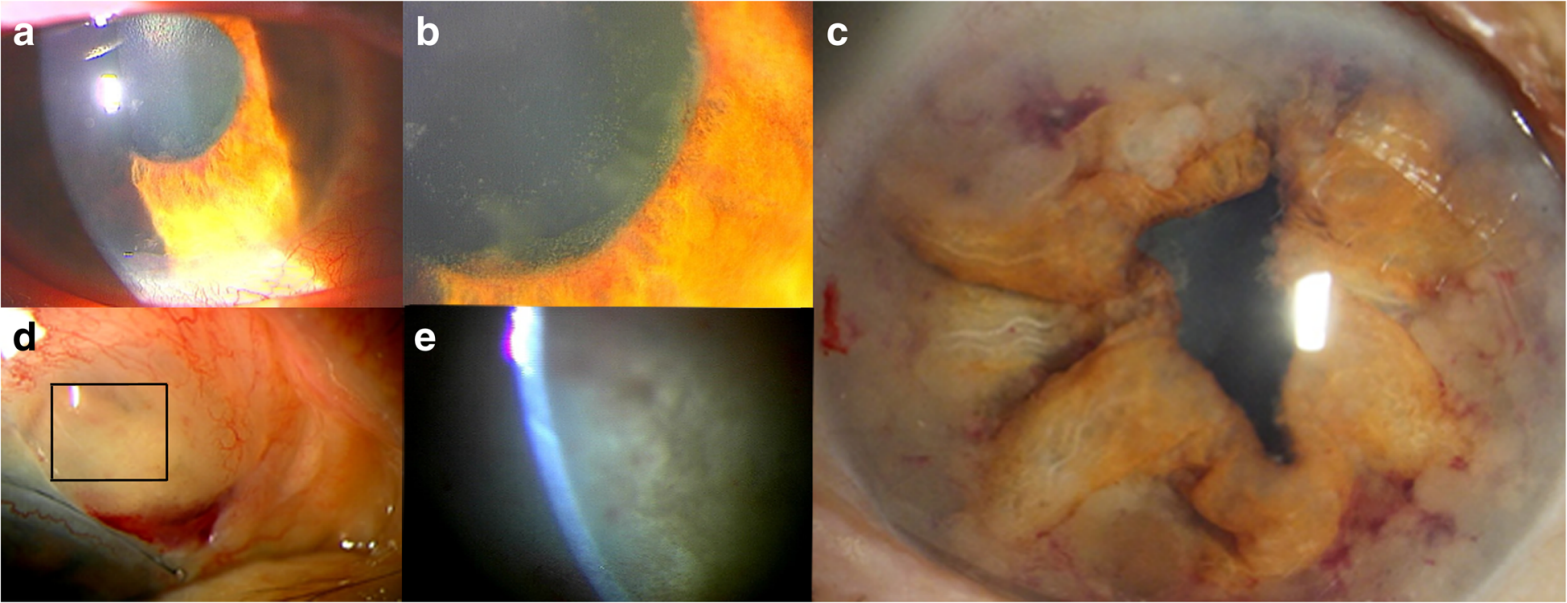
Slit-lamp photograph of the right eye. **a**: Slit-lamp images showing fibrin-like membrane on the iris, ciliary injection, and iris neovascularization around pupil without corectopia at initial visit. **b**: Magnified image of a. **c**: Slit-lamp image showing white, frog spawn-like mass with corectopia and hemorrhage in the anterior chamber 2 months after trabeculectomy. Iris appears atrophic and partially swollen. **d**: Slit-lamp image showing filtering bleb with hemorrhage and many white, frog spawn-like mass 2 months after trabeculectomy. **e**: Magnified image of d

A 74-year-old Japanese woman visited our hospital in 2016 with visual disturbance and uncontrolled intraocular pressure (IOP) in her right eye. She was diabetic and hypertensive. She had no history of systemic cancer, malignant lymphoma, or ocular manifestations of cancer. Her best-corrected visual acuity (BCVA) was 20/100 OD and 20/200 OS, and her manifest spherical refractive error was − 10.0 diopters OU. The IOP was 28 mmHg OD and 12 mmHg OS. Slit-lamp examination showed ciliary injection of the conjunctiva, clear and smooth cornea, strong cortical cataract, fibrin-like membrane without a mass on the iris, iris neovascularization without posterior synechia, and infiltration of inflammatory cells (Fig. 1a and b). Gonioscopy demonstrated neovascularization of the angle but no hypopyon, nodules, or peripheral anterior synechia. Ophthalmoscopy and fundus photography revealed no vitreous opacifications, retinal exudates, and retinal hemorrhages. The left eye was normal except for a cataract and retinoschisis with high myopia. Hematologic examinations showed increased blood sugar (194 mg/dl) and decreased platelet count (12.2 × 10^4^/μl).

She was initially diagnosed with acute iritis secondary to diabetes mellitus which was partly based on her history and fibrin-like membrane on the surface of the iris in the right eye. The acute iritis and secondary glaucoma were treated with topical steroids and anti-glaucoma medications. The elevated IOP in right eye did not respond to the medications. Thus 1 month after the initial visit, trabeculectomy with mitomycin C and peripheral iridectomy was performed. After the trabeculectomy, the IOP in the right eye was decreased to around 10 mmHg. However, two months after the trabeculectomy, she presented with a bulging iris, and white, frog spawn-like mass resembling fibrin in the right anterior chamber (Fig. 1c) and in the trabeculectomy bleb (Fig. 1d and e). The BCVA in the right eye was 20/100 and the IOP was 17 mmHg.

**Fig. 2:**
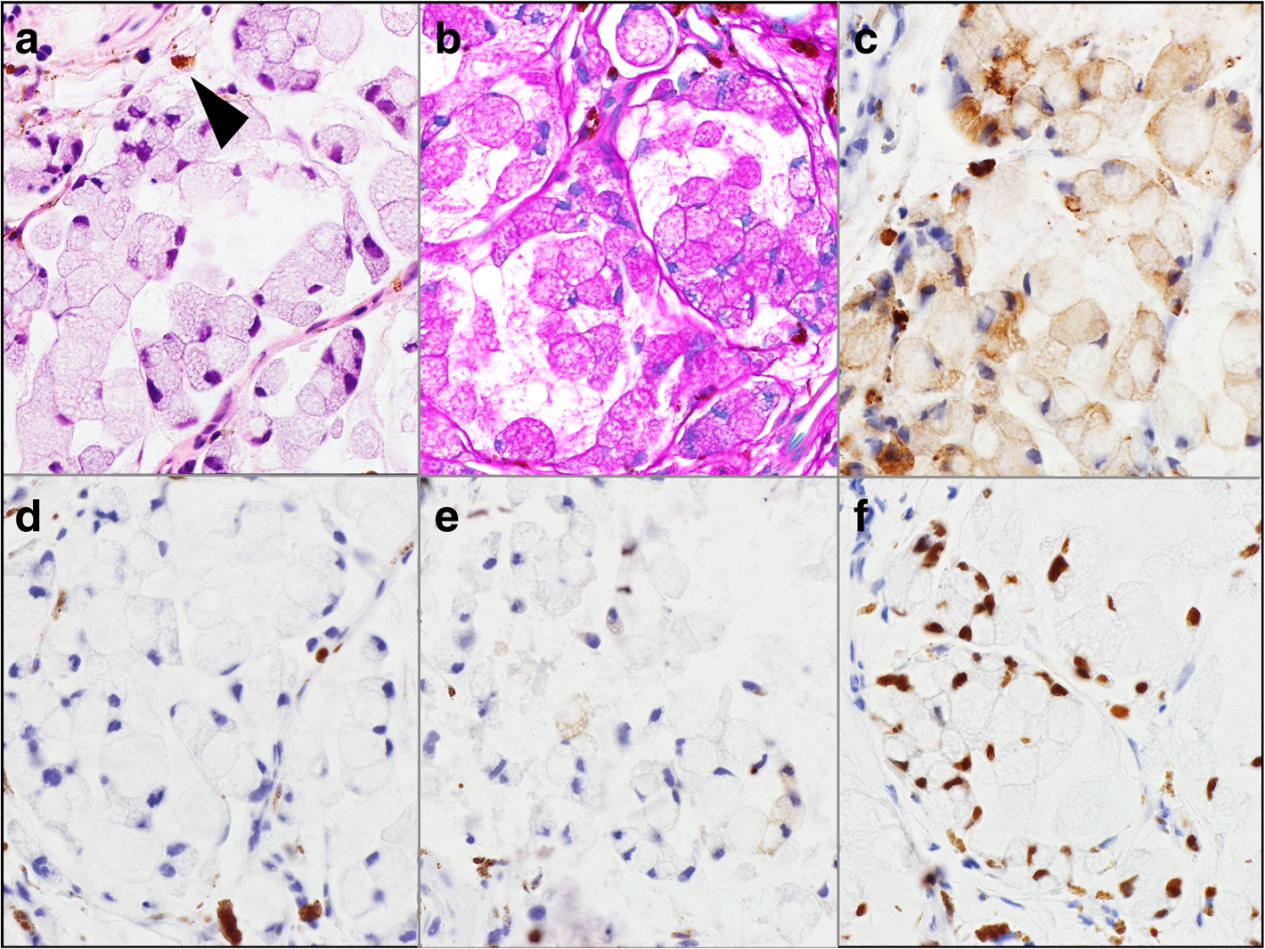
Representative photomicrograph showing tumor cells infiltrated onto the iris. H&E stain. PAS stain, and immunostaining. **a**: Hematoxylin and eosin (H&E) stain photomicrographs showing signet ring-like tumor cells and melanin containing-cells (arrowhead) in the interstitium of iris. **b**: Periodic acid-Schiff (PAS) stained section showing tumor cells containing a large amount of mucin. **c**: Immunostaining showing positivity for CK-CAM5.2. **d**:, **e**:, and **f**. Immunostaining showing negativity for CK7 and CK20, and positivity for CDX2 indicating enteric differentiation of the tumor cells, and primary tumor location might be gastrointestinal tract

A malignant lymphoma was suspected based on the white, frog spawn-like mass resembling fibrin which did not respond to the steroid application. Six months after the trabeculectomy, we then performed fine needle aspiration biopsy for cytological diagnosis. The cytological results demonstrated foamy histiocyte-like cells with mild nuclear atypia but no signs of malignant lymphoma. Thus iris biopsy was performed. The biopsy specimen showed an atrophic and fragile iris, and pathological examination suggested metastatic adenocarcinoma with intracytoplasmic mucin and signet ring cell features (Fig. 2). Immunostaining for CK-CAM5.2, and CDX2 were positive and CK7 and CK20 were negative indicating enteric differentiation of the tumor cells, and primary tumor location might be gastrointestinal tract (Fig. 2).

Examination by computed tomography, magnetic resonance imaging, positron emission tomography, and esophagogastroduodenoscopy revealed advanced gastric cancer with peritoneal and lymph nodes metastasis and ascites. She was treated with combination chemotherapy of Tegafur/gimeracil/oteracil (S-1) and cisplatin, also known as cis-diamine dichloroplatinum (CDDP), for the gastric cancer. However, the white, frog spawn-like mass in the anterior chamber was not resolved. At the final visit, the BCVA in the right eye had worsened to light perception, and the IOP was 48 mmHg. The left eye did not have any mass lesion and the BCVA was 20/66 because of retinoschisis with high myopia. She died from pneumonia at 8 month from initial visit.

## Discussion

The manifestations of an iris metastatic tumor are usually a white or yellow colored mass on the iris [2, 3]. Two case reports of iris metastatic tumors from gastric cancers have been reported; one had a white lace-like avascular mass on the iris, and the other had a yellowish-white vascularized mass on the iris [2, 3]. Our case had a white, frog spawn-like mass that resembled fibrin rather than lace-like and a vascularized mass. We suggest that the gastric signet ring cells had metastasized to the iris stroma causing the bulging of the atrophic iris in our case. The earlier case reports did not mention a bulging atrophic iris [2, 3].

The main symptoms and signs of iris metastasis in the 107 eyes of 104 patients reported by Shields et al. were eye pain, blurred vision, secondary glaucoma, and corectopia [4]. Another study reported corectopia, prominent epibulbar injection, and secondary glaucoma [5]. The authors concluded that the features of iris metastasis were generally distinctive enough to differentiate them from other intraocular neoplasms and inflammations [5]. Our case had blurred vision, epibulbar injection, and secondary glaucoma at the initial examination. These findings are not specific for iris metastasis, thus a diagnosis of iris metastasis was not made. A history of systemic tumors and typical mass on the iris are important signs for a diagnosis of iris metastasis. However, 32% of patients with iris metastasis have ophthalmic features that precede the diagnosis of the systemic tumors [5]. In addition, iris metastasis can have a broad spectrum of clinical presentations [5].

## Conclusion

We present a case of a metastasis of a gastric adenocarcinoma to the iris that was detected before a diagnosis of gastric signet ring cell adenocarcinoma. Our findings indicate that patients with acute iritis with an atypical iris mass resembling fibrin and secondary glaucoma should be examined comprehensively for systemic tumors.

## Abbreviations

BCVA: Best-corrected visual acuity
CDDP: Cis-diamine dichloroplatinum
H&E: Hematoxylin eosin
IOP: Intraocular pressure
PAS: Periodic acid-Schiff
